# Impact of Rapid Eye Movement Sleep Deprivation on Pain Behaviour and Oxidative Stress in the Thalamus: Role of Tualang Honey Supplementation

**DOI:** 10.21315/mjms2022.29.2.7

**Published:** 2022-04-21

**Authors:** Mohd Shafie ANIS SYAHIRAH, Abd Aziz CHE BADARIAH, Long IDRIS, Siran ROSFAIIZAH, Noordin LIZA

**Affiliations:** 1 Department of Physiology, School of Medical Sciences, Universiti Sains Malaysia, Kelantan, Malaysia; 2 School of Health Sciences, Universiti Sains Malaysia, Kelantan, Malaysia; 3 Neuroscience Research Group, Faculty of Medicine, Universiti Teknologi Mara, Selangor, Malaysia

**Keywords:** REM sleep deprivation, Tualang honey, pain behaviour score, oxidative stress biomarkers

## Abstract

**Background:**

Insufficient sleep alters the body’s physiological functions. This study investigated whether oxidative stress (OS) in the thalamus was correlated with the pain behaviour score in the rapid eye movement (REM) sleep-deprived rat model.

**Methods:**

Four groups of Sprague-Dawley rats were included in the study (*n* = 6): i) control; ii) REM sleep-deprived rats for 72 h (REMsd); iii) REM sleep-deprived rats for 72 h pretreated with Tualang honey (REMsd-H) and iv) tank control (TC). Following the intervention, 1% formalin was injected on the right hind paw and pain behaviour was recorded for 1 h. OS markers of glutathione (GSH), glutathione reductase (GR), superoxide dismutase (SOD), catalase (CAT) and malondialdehyde (MDA) in the thalamus were measured.

**Results:**

REM sleep deprivation increased pain behaviour scores in phase II of the formalin test with decreased GSH, GR, SOD and CAT. The MDA level was significantly higher in the REMsd compared to REMsd-H. There was an inverse correlation between pain behaviour scores and GSH, GR and SOD levels. A positive correlation was found between the pain behaviour score and the MDA level.

**Conclusion:**

OS levels were significantly correlated with the pain behaviour score in the REMsd rats. Tualang honey administration reduced pain behaviour score and OS in the thalamus.

## Introduction

Sleep has a vital role in life, and it is involved in growth, neurotransmitter synthesis in the central nervous system, and modulation of immune and hormonal responses. Insufficient sleep may lead to difficulties in concentration, vision disturbances, slower reactions, poor memory (1) and may alter pain responses. The relationship between sleep disturbance and pain is bidirectional; pain can affect sleep quality and sleep disturbance can influence pain intensity. Human studies have shown that sleep deprivation is associated with increased pain perception (2–3). The severity of pain is affected by the degree of sleep disturbance in humans (4) and animals (5).

Potential mechanisms responsible for the alteration of pain responses following sleep disturbances might be related to the modulation of nociceptive pathways (e.g. opioid, monoaminergic and gamma amino butyric acid (GABA)ergic systems). Furthermore, hyper-reactivity of the hypothalamic-pituitary-adrenal axis to stressors (6) with upregulation of inflammatory mediators in various types of pain can be seen associated with sleep disturbances (7–8). Besides, sleep disturbance may cause an imbalance between the oxidant and antioxidant systems, resulting in oxidative stress (OS) (9). Although several studies demonstrated the association of OS with the degree of pain (10–11), its role in modulating pain responses in the central nervous system following sleep deprivation is not known.

Previous studies have shown that OS occurred in the hippocampus (12), hypothalamus, midbrain and hindbrain of rats (13) following rapid eye movement (REM) sleep deprivation. It is unknown whether OS occurs in pain modulating structures, such as the thalamus following REM sleep deprivation. If OS occurs in the thalamus, there is a correlation between the parameters and the pain behaviour score. The thalamus has been shown to modulate nociceptive responses in electrophysiological and imaging studies following thermal stimulation, inflammation, nerve injury and clinical pain (14–18).

An antioxidant can reduce OS by interrupting the propagation of free radicals or by inhibiting the formation of free radicals (19). Various types of antioxidants have been attributed to reducing OS such as vitamins C and E and glutathione (GSH) (20–21). In recent years, Tualang honey has been investigated for its potential therapeutic effects. Tualang honey is rich in phenolic acids and flavonoids. The phenolic components consist of gallic, syringic, benzoic, trans-cinnamic, p-coumaric and caffeic acids, whereas the flavonoid components consist of catechin, kaempferol, naringenin, luteolin and apigenin (22). Besides containing antioxidants, it has been reported to possess antimicrobial, anti-inflammatory and antitumor properties (23). Most recently, we have demonstrated that the administration of Tualang honey could reduce the pain behaviour score, improve the morphology of neurons, and reduce OS and N-methyl-D-aspartate (NMDA) levels in the thalamus in the offspring of prenatally stressed rats (24). Thus, it remains to be elucidated whether Tualang honey administration could provide a protective effect against the alteration of the thalamus due to REM sleep deprivation. The present study aimed to elucidate the impact of REM sleep deprivation on OS parameters and whether the parameters are correlated with the pain behaviour score in the formalin pain model. If the parameters are correlated, the administration of Tualang honey, as an antioxidant, is beneficial to reduce the impact of REM sleep deprivation.

## Methods

### Animal Study

Eight to ten weeks old Sprague-Dawley male rats (180 g–200 g) were bought from the Animal Research and Services Centre (ARASC), Universiti Sains Malaysia. The rats were divided into four groups (*n* = 6 per group): i) control; ii) REM sleep-deprived rats for 72 h (REMsd); iii) REM sleep-deprived rats for 72 h pretreated with Tualang honey (REMsd-H), and iv) tank control (TC) rats. Tualang honey (1.2 g/kg body weight) was the antioxidant used in the study and it was given by oral gavage for 4 weeks before adaptation. Rats were given standard laboratory food and water.

### Adaptation Period

For adaptation, each rat in REMsd, REMsd-H and TC groups was adapted individually in a dry tank with two inverted flowerpots (25–26), while each rat in the control group was placed individually in a standard dry cage for 72 h. The size of the tank used was 30 cm height, 30 cm length and 60 cm width. For REMsd and REMsd-H groups, two flowerpots (6.5 cm diameter) were placed in the dry tank. Meanwhile, a wider-diameter of flowerpot (13.5 cm diameter) was used for the TC group. To reduce stress due to social isolation, two rats underwent adaptation concurrently by placing two dry tanks side by side. The adaptation period was necessary so that the rats were adapted to the surroundings in the glass tank.

### REM Sleep Deprivation Model

The glass tanks were filled with water up to 1 cm from the top of the platforms (6.5 cm diameter) in the glass tank. The water temperature was maintained at 30 ˚C. When the rats fell asleep, the muscles became atonic during the REM phase and they would fall into the water. The water prevented the rats from sleeping and the rats would get back on the platform. The inverted flowerpot technique had been utilised by other studies to achieve REM sleep deprivation (25–28). Food and drinks were provided throughout the adaptation and experiment.

### Control Groups

Each rat in the control group was placed in a cage with 22 cm in height, 56 cm in length and 38 cm in width. The cages were placed side by side to minimise social isolation stress. The glass tank for the TC group was also filled with water up to 1 cm from the top of the platform. The platforms used were larger in diameter (13.5 cm diameter) compared to REMsd and REMsd-H groups. The larger platforms were provided to ensure that this group of rats would be not REM sleep-deprived while examining the effect of exposure to the aquatic environment (25–26).

### Formalin Test

The formalin test was chosen because it is considered a very powerful tool in pre-clinical research (29). Furthermore, this test allows the study of two different kinds of pain, i.e. acute peripheral pain mediated by the direct activation of nociceptors, and inflammatory and central nociceptive sensitisation (30).

Before formalin injection, each rat was acclimatised in a Perspex observation chamber (26 cm × 20 cm × 20 cm) for 15 min. One percent of formalin (50 μL) was given by subcutaneous injection on the right hind paw. The pain behaviour was recorded for 60 min and assessed later. Two hours following formalin injection, the rats were sacrificed using intraperitoneal injection of sodium pentobarbitone (31) followed by decapitation using a guillotine. The pain behaviour score was tabulated for every minute and the scores were averaged every 5 min. The pain behaviour score was given from 0 to 3 according to the scoring system as described below (31):

- Score 0 (absence of pain): The injected hind paw was placed normally on the floor.
- Score 1 (mild pain): The hind paw rested lightly on the floor or the rat walked with a limping gait.
- Score 2 (moderate pain): The hind paw was raised without touching any surface.
- Score 3 (severe pain): The hind paw was licked, bitten and shaken.

In this study, the average score from minute 0 to 10 represented the pain behaviour score for phase 1. The average score from minute 15 to 35 represented the early phase 2, whereas the average score from minute 40 to 60 represented the late phase 2 of the formalin test (32).

### Brain Removal and Harvesting

The brain samples were harvested and weighed using a digital analytical balance. The thalamus was identified using rat brain atlas (33) and fixed in 0.1 M phosphate-buffered saline (PBS, pH 7.4). Each brain was then homogenised in ice-cold PBS (10% w/v) and kept at −80 ˚C in individual tubes.

### Quantification of Oxidative Stress Parameters

OS parameters in the thalamus were evaluated with the measurement of levels of GSH, glutathione reductase (GR), superoxide dismutase (SOD) and catalase (CAT) using ELISA kits from Bioassay (Bioassay System, Hayward, USA). Meanwhile, the levels of malondialdehyde (MDA) were measured using ELISA kits from Northwest (Northwest Life Science, Vancouver, Canada) ELISA kits.

### Statistical Analysis

Normality test was performed to check the distribution of data before statistical analysis. Data entry and analysis were done using GraphPad Prism version 8.00. The pain behaviour score was analysed with repeated-measure analysis of variance (ANOVA) followed by post-hoc Bonferroni test. One-way ANOVA was used to compare the differences between OS parameters in the thalamus. The differences in the parameters between the groups were considered significant when *P* < 0.05. The data in this study are expressed as mean (standard deviation [SD]). Correlation analysis was conducted to determine the relationship between OS parameters and mean pain behaviour score. The strength of the correlation is indicated by the Pearson correlation coefficient (*r*), where *r* < 0.25 indicates a poor correlation, *r* between 0.26 and 0.50 indicates a fair correlation, *r* between 0.51 and 0.75 indicates a good correlation and *r* between 0.76 and 1.00 indicates an excellent correlation.

## Results

### Formalin Test

**Figure 1:**
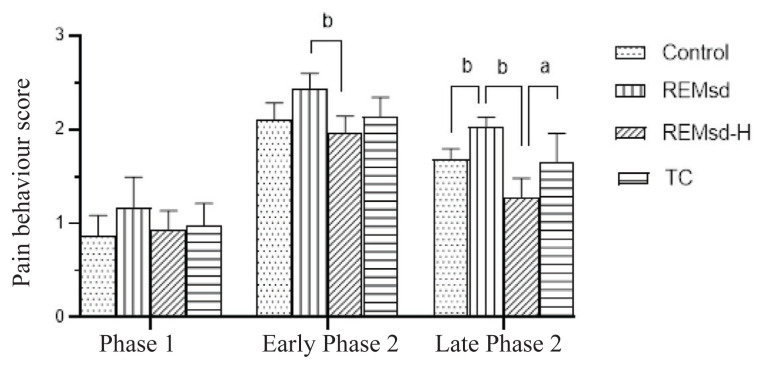
Effects of REM sleep deprivation and Tualang honey administration on pain behaviour score in each phase of formalin test (*n* = 6). REM sleep deprivation has significantly increased pain behaviour score in late phase 2 (^b^*P* < 0.001) compared to Control. There were significant differences when compared between REMsd-H and REMsd groups during early and late phase 2 (^b^*P* < 0.001). The difference was also significant when compared between REMsd-H and TC groups (^a^*P* < 0.01).

There was a significant difference between time and group interaction (*F*(6, 70) = 4.762; *P* < 0.001). The analysis on the pain behaviour score by one-way ANOVA demonstrated significant differences in early (*F*(3, 36) = 11.87; *P* < 0.001) and late phase 2 (*F*(3, 36) = 23.06; *P* < 0.001) when compared between all groups (Figure 1). The differences in phase 1 were insignificant. REM sleep deprivation was associated with increased pain behaviour score when compared to the control group in early and late phase 2; however, it was only significant in late phase 2 (*P* < 0.001). A significant difference was also seen when compared between REMsd and TC groups (*P* < 0.05). Oral administration of Tualang honey was associated with a reduction in the pain behaviour score in early and late phase 2 compared to the REMsd group (*P* < 0.001).

### Level of Oxidative Stress Parameters

The differences in the level of GSH (F(3, 20) = 24.76), GR (F(3, 20) = 16.62), SOD (F(3, 20) = 62.23), CAT (F(3, 20) = 23.13) and MDA (F(3, 20) = 67.19) were found to be significant when compared between the groups (*P* < 0.05). The REMsd group showed a lower level of antioxidants (GSH, GR, SOD and CAT) and a higher level of MDA (Table 1) compared to other groups. Administration of Tualang honey was associated with a significantly higher level of GSH, GR, SOD and CAT, and a significantly lower level of MDA compared to the REMsd group (*P* < 0.001).

### Correlation Study

**Figure 2:**
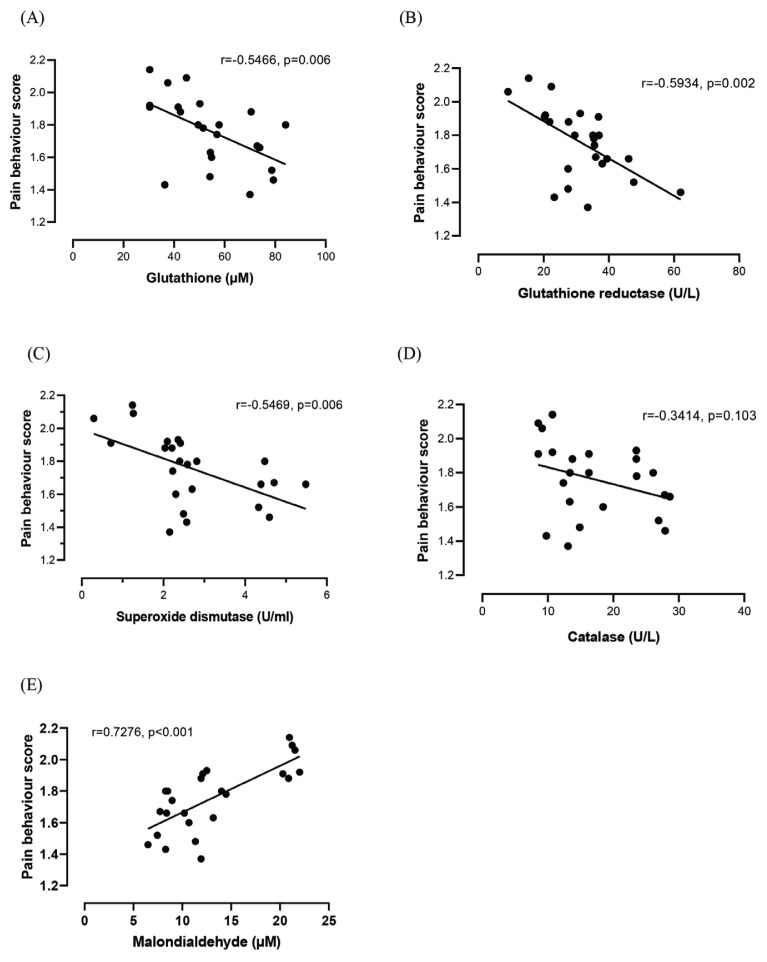
Scatter plots of correlations between OS parameters: A) glutathione, B) glutathione reductase, C) superoxide dismutase, D) catalase and E) malondialdehyde and pain behaviour score

Based on Pearson correlation (Figure 2), GSH, GR and SOD showed a good inverse correlation, whereas CAT showed a fair inverse correlation with the pain behaviour score. Meanwhile, MDA showed a good positive correlation with the pain behaviour score.

## Discussion

The pain in phase 1 of the formalin test was contributed by formalin-induced inflammation in the periphery (34), whereas the pain in phase II was due to changes in the central nervous system as well as the persistence of peripheral-induced inflammation. A report has shown that prednisolone (glucocorticoid) significantly reduced the pain behaviour score in early phase 2 (32) and it was suggested that the inhibition was mediated by annexin 1, which reduced spinal prostaglandin E2 (35).

Sleep disturbance has been linked to increased pain responses in animal and human studies (2–5, 36). In this study, the group that was given Tualang honey showed a reduction of pain behaviour score in phase 2, but the reduction was only significant in late phase 2. Kinin and other inflammatory mediators were shown to be released following formalin-induced inflammation in the periphery. The components of honey, including catechin and chlorogenic acid, were reported to inhibit kinin and other inflammatory mediators released peripherally (37–38), and this mechanism might contribute to the pain suppression in the Tualang honey group.

Another report has demonstrated that Tualang honey reduced inflammation in the central nervous system by decreasing the levels of TNF-α, IL-1, glial fibrillary acidic protein, allograft inflammatory factor 1 and COX-2 in the rats (39). A report has shown that the termination of pain behaviour at the later part of phase 2 was contributed by an endogenous inhibitory mechanism, which was not mediated by opioid receptors (40). There is a possibility that Tualang honey facilitates the inhibitory mechanism to reduce the pain behaviour score in late phase 2 of the formalin test. In this study, Tualang honey administration in the REMsd-H group was associated with decreased pain behaviour scores in phase 2 and not phase 1 of the formalin test. This suggests the central action of Tualang honey in modulating the pain responses, and in this study, we have chosen the thalamus as it is one of the structures in the pain pathway. Although increased pain behaviour in phase 2 may be contributed by modulation at the peripheral or central nervous system level, imaging studies have shown the altered activity in the thalamus following sleep deprivation (41–42).

In addition, a report has demonstrated that REM sleep deprivation increased the sensitivity to mechanical stimuli, with increased Fos protein in brain areas involved in nociceptive transmission (e.g., hypothalamus, periaqueductal gray and trigeminocervical complex (43). Several studies have demonstrated the presence of OS following sleep deprivation (9, 13). A few reports have shown the association of OS with the degree of pain (10, 11); however, its role in modulating pain is not much known. Furthermore, changes in OS parameters have not been reported in the thalamus following REM sleep deprivation. In this study, antioxidants GSH, GR, SOD and CAT were markedly reduced, while the MDA level was increased in the REMsd group.

Other studies have reported that OS contributes to the modulation of pain in various pain models [e.g. prenatal stress (44), cancer pain model (45), neuropathic (46) and arthritic pain (47)]. Accumulated reactive oxygen species (ROS) might contribute to increased neuronal excitability by reducing inhibitory transmission of GABAergic neurons in the spinal dorsal horn (48). However, ROS may reduce GABAergic transmission but did not decrease glycine transmission (48). With the persistence of peripheral inflammation, synaptic activity and neurotransmitter release (e.g. glutamate and glycine), increased. The binding of the neurotransmitters to NMDA receptors will modulate the nociceptive pathways leading to increased pain responses (49).

The activation of neuronal NMDA receptors has been shown to induce the release of superoxides-mediated OS in neighbouring neurons and astrocytes (50). The NMDA receptors are responsible for regulating the intracellular calcium level in the neurons. The mechanism for the increase in OS markers is believed to be due to calcium dysregulation as a result of NMDA receptor hyperactivity, which leads to neurotoxicity (51). In this study, Tualang honey administration was associated with a reduction in the pain behaviour score with higher antioxidant and lower MDA levels in the thalamus. Tualang honey is a natural product with a high concentration of phenolics and flavonoids that have important anti-inflammatory and antioxidant activities (32, 52–55). Sleep deprivation causes depletion of antioxidants and increases radical oxygen species that modified the membrane function and eventually damaged the cells in the brain, resulting in reduced neuronal number and abnormal histological features (55). The present study has also shown that the level of antioxidants in the thalamus is inversely correlated with the pain behaviour score, whereas the oxidant (MDA) is positively correlated with the score. These results suggest that OS in the thalamus contributed to the pain modulation following REM sleep deprivation.

Roles of signalling pathways in chemical-induced hyperalgesia have been reported. Extracellular signal-regulated kinase (ERK) is an important molecule in mitogen-activated protein kinase signalling pathways, which is known to play an essential role in the inflammatory pain. Furthermore, ERK can be phosphorylated (p-ERK) in the presence of noxious substances that stimulate sensory neurons (56). Previous studies in mice have shown increased expressions of p-ERK in the ipsilateral dorsal horn of lumbar spinal cord in formalin-induced (57) and complete Freund’s adjuvant-induced pain (58). Meanwhile, carrageenan-induced hyperalgesia in mice was demonstrated to involve nuclear factor kappa B (NF-κB) signalling pathway activation, which induces OS and cytokine production peripherally in the paw skin and centrally in the spinal cord (59). The carrageenan-induced animal pain model has been linked to defective cellular antioxidant systems, including SOD, CAT and glutathione peroxidase in the skin tissue oedema (60). This study demonstrated decreased antioxidants and increased MDA levels in the thalamus of REMsd rats. Thus, our next step is to evaluate the role of OS in the inflammatory response in the paw tissue induced by formalin injection.

## Conclusion

In conclusion, REM sleep deprivation was associated with increased pain behaviour score and OS in the thalamus. The antioxidants in the thalamus showed a fair to good inverse correlation with the pain behaviour score, while MDA demonstrated a good correlation with the score. Tualang honey supplementation has reduced OS in the thalamus of REMsd rat models and thus modulated the pain behaviour in the formalin test.

## Figures and Tables

**Table 1:** Level of OS parameters in the experimental groups

OS parameters	Control mean (SD)	REMsd mean (SD)	REMsd-H mean (SD)	TC mean (SD)
GSH (μM)	54.16 (3.16)	36.02 (6.62)^a^	77.09 (4.41)^b^	53.85 (14.19)
GR (U/L)	31.78 (4.01)	18.24 (5.12)^a^	44.69 (9.73)^b^	31.76 (5.61)
SOD (U/mL)	2.47 (0.21)	1.27 (0.71)^a^	4.66 (0.42)^b^	2.40 (0.21)
CAT (U/L)	16.49 (4.07)	10.23 (1.98)^a^	27.67 (0.99)^b^	16.59 (5.75)
MDA (μM)	11.30 (2.54)	21.13 (0.59)^a^	8.13 (1.25)^b^	11.65 (1.71)

Notes: Data are presented as mean (SD), *n* = 6 rats in each group;

a *P* < 0.05 when compared to REMsd-H;

b *P* < 0.05 when compared to Control and TC groups
